# Hinderin, a five-domains protein including coiled-coil motifs that binds to SMC3

**DOI:** 10.1186/1471-2121-6-3

**Published:** 2005-01-18

**Authors:** Chirag A Patel, Giancarlo Ghiselli

**Affiliations:** 1 Department of Pathology and Cell Biology, Thomas Jefferson University, Philadelphia, 19107, USA; 2 Kimmel Cancer Center, Thomas Jefferson University, Philadelphia, 19107, USA

## Abstract

**Background:**

The structural maintenance of chromosome proteins SMC1 and SMC3 play an important role in the maintenance of chromosomal integrity by preventing the premature separation of the sister chromatids at the onset of anaphase. The two proteins are constitutive components of the multimeric complex cohesin and form dimers by interacting at their central globular regions.

**Results:**

In order to identify proteins that by binding to SMC3 may interfere with the protein dimerization process, a human cDNA library was screened by the yeast two-hybrid system by using the hinge region of SMC3 as bait. This has lead to the identification of Hinderin, a novel five domains protein including two coiled-coil motifs and sharing a strikingly structural similarity to the SMC family of proteins. Hinderin is ubiquitously expressed in human tissues. Orthologue forms of the protein are present in other vertebrates but not in lower organisms. A mapping of the interaction sites revealed that the N- and C-terminal globular domains mediate the binding of Hinderin to SMC3. Hinderin/SMC3 complexes could be recovered by immunoprecipitation from cell lysates using an anti-SMC3 antibody, thus demonstrating that the two proteins interact in vivo. On the contrary, Hinderin did not interact with SMC1. In vivo the rate of SMC1/SMC3 interaction was decreased by the ectopic expression of Hinderin.

**Conclusions:**

Hinderin is a novel binding partner of SMC3. Based on its ability to modulate SMC1/SMC3 interaction we postulate that Hinderin affects the availability of SMC3 to engage in the formation of multimeric protein complexes.

## Background

The structural maintenance of chromosome (SMC) proteins are involved in several aspects of chromosomal dynamic, in DNA recombination and in DNA repairs [1-3]. Two SMC proteins named SMC1 and SMC3 bind to and prevent the premature separation of sister chromatids at the end of mitosis [4,5]. SMC1 and SMC3 directly interact through their central globular binding domains by forming an heterodimer [6,7]. The protein complex encircles the sister chromatids and is stabilized though the interaction with two other cohesin proteins known as Scc1 and Scc3 in s. cereviasie [7,8]. At anaphase, the ring-shaped complex is broken down when separase, a cysteine protease, cleaves Scc1, thus freeing the sister chromatids to move in opposite directions [6,9]. Somatic cells with deranged separase activity or lacking Scc1 develop aneuploidy at increased rate. This suggests that the cohesin complex plays a major role in the maintenance of chromosomal stability [10-13]. The mechanism regulating the interaction between SMC1 and SMC3 is still poorly understood. It has however been established that a single point mutation of the central globular domain (known as hinge) of either one of these proteins strongly affects the dimerization rate and prevents the attachment of the cohesin complex to chromatid DNA [7]. Conceivably, proteins that interact with the hinge domain of SMC1 or SMC3 can act as modulator of the cohesin complex formation and may thus affect chromosomal stability. In this paper we report the identification of a new SMC3-interacting protein that specifically binds to SMC3's central globular domain. The sequence of the identified gene product matched that of a previously discovered hypothetical new protein with no known function. We have named the protein Hinderin. The gene is expressed in all the human tissues analyzed thus far. Orthologue forms of this protein are expressed in vertebrates but not in lower organisms. Hinderin is a five-domain proteins and its structure resembles that of SMC proteins with N- and C-terminal globular domains that are joined by a coiled coil region interrupted at the center by a third globular domain. However, unlike the canonical SMC proteins, Hinderin does not harbor ABC-like ATPase sequences. We have found that the protein interacts with the hinge region of SMC3 but not with SMC1. Hinderin acts as a binding competitor of SMC1 and, as such, qualifies as a putative modulator of the SMC3 function.

## Results

### Identification of Hinderin, an SMC3-interacting protein with five domain structure including coiled-coil motifs

The region of SMC3 encompassing the protein hinge domain (Gln^465 ^to Gln^807^) was used as bait in a yeast two-hybrid system to identify interacting proteins expressed by a human fetal brain Matchmaker two-hybrid cDNA library (Clontech). About 3 × 10^6 ^library clones were screened. Forty blue colonies reaching 2 mm in size after one week were collected and 21 of the isolated plasmids with inserts greater than 500 bp were sequenced. Three of the sequences matched the same region of the published cDNA Genbank clones AB037749 (coding for the hypothetical protein KIAA1328) and AL832625 (corresponding to the hypothetical protein DKFZp451C1618). The inserts of ~2 kb included part of the gene 3'-UTR. However the 5'-end of the coding region was not present in the retrieved clones. The issue was complicated by the fact that the sequences of AB037749 and AL832625 diverged at their 5'-end. 5'-RACE was thus employed to identify the transcriptional start site of the interacting gene by using mRNA derived from fetal kidney 293, hepatoma HepG2, and cervical HeLa human cells. All the cloned sequence coincided with that of the AL832625 clone and matched in full the putative coding sequence obtained by automated computational analysis of the human genome (Genbank XM029429). The conceptually translated sequence coded for a protein of 578 amino acids.

**Figure 1:**
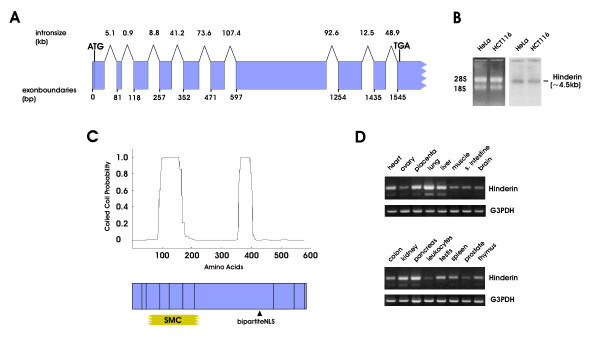
**Hinderin: Genomic organization, structural domains, and expression pattern in human tissues. **A) The sequence of the *Hinderin *ORF was used to BLAST the human genome database. The numeration of the exon boundaries is relative to the transcriptional start site. The size of the intronic sequences is based on the numeration of the human contiguous sequences. B) Northern blot hybridization of *Hinderin *in HeLa and HCT116 cells. A single main transcript of ~ 4.5 kb is identifiable. C) Prediction of coiled-coil domains in Hinderin. The numbers on the abscissa corresponds to the amino acid residues. The probability of the polypeptide to assume a coiled coil conformation is plotted on the ordinate axis. The contribution of the different exons to the five-domain structural organization of Hinderin is also illustrated. The sequence encoded by exons 3 through 6, bears a 36% homology to the SMC protein consensus sequence. A bipartite nuclear localization signal is harbored in exon 7. D) Expression of *Hinderin *in different human tissues. PCR amplification was stopped after 30 cycles and the product analyzed on 2% agarose staining with ethidium bromide. The *G3PDH *transcript was amplified in 20 cycles and used to show uniformity of the source cDNA in the different specimens.

The exam of the secondary structure of the protein revealed a remarkable similarity with the structural organization of SMC proteins, particular with regard to the five-domain structure typical of the SMC protein family. The protein features C- and N-terminal globular domains, joined by a coiled-coil sequence interrupted in the middle by a third globular domain. The size of the central globular domain is similar to that of SMC3 and SMC1 but the remaining domains are smaller in size. Based on its ability to interact with the hinge domain of SMC3 and thus to potentially affect SMC3/SMC1 dimerization, the new protein was named Hinderin. The *Hinderin *gene spans ~400 kb of the human genome and is located on chromosome 18p11-2. The coding sequence is organized in 10 exons (fig. 1A). Exon 10 is the largest and contains an extended 3'-UTR. Northern blot hybridization analysis performed on mRNA from HeLa and colon carcinoma HCT116 human cell lines, showed a single major transcriptional product of ~4.5 kb (fig. 1B). A correlation between the exonic organization and the protein structural motifs was apparent (fig. 1C). The N-terminal globular domain is encoded by exons 1–3. The first coiled-coil domain is encoded by exons 4–6. Exon 7 harbors the entire central globular domain, the central coiled-coil region, and part of the C-terminal globular domain. The polypeptide encompassing exons 3 to 6 displays a 36% sequence homology to the consensus sequence of SMC family of proteins. A bipartite nuclear localization signal (R^441^KERK) is located in exon 7 within a coiled-coil region. The Hinderin expression pattern in a panel of 16 human tissues was analyzed by semiquantitative RT-PCR (fig. 1D). The results indicate that the gene is expressed ubiquitously with the highest expression level detected in the lung, liver, placenta, kidney, and pancreas. The lowest expression level was detected in leukocytes and the prostate.

### Mapping of the Hinderin and SMC3 interaction domains

**Figure 2:**
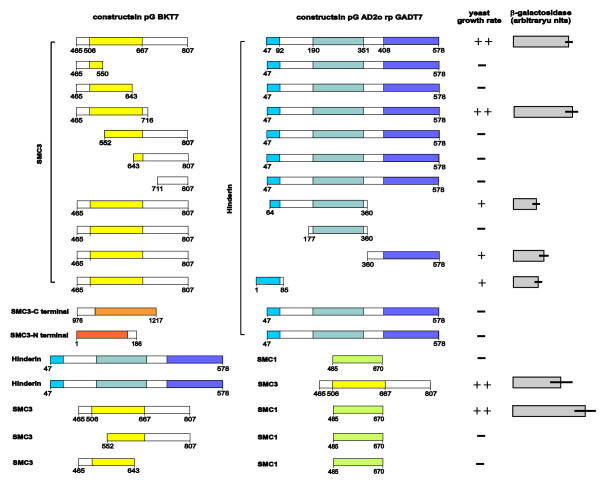
**Yeast two-hybrid assay of the interaction of different regions of SMC3 and SMC1 with Hinderin. **A) SMC3 and Hinderin constructs in pGBKT7 are schematized on the left-hand side. All numerals refer to the amino acid sequence. The SMC3-465/807 construct harbors the protein hinge domain and was utilized as bait for the screening of the Clontech Matchmaker human cDNA library. SMC1, SMC3 and Hinderin constructs cloned in pACT2 or pGADT7 are illustrated on the right-hand side of the panel. H-47/577 represents the pACT2 clone retrieved from the screening. We scored the strength of interaction based on the rate of appearance of Blue colonies and the intensity of the color developed. The null score (-) was assigned when no blue colonies were visible after ten days. To obtain a quantitative measure of the rate of protein interaction, yeast colonies were grown in selective media and after lysis the β-galactosidase activity released in the supernatant assayed using a chromogenic substrate as detailed in the Methods. The results shown are the mean ± SD of three independent determinations.

In order to map the region of Hinderin interacting with SMC3, AH109 yeast was cotransformed with the SMC3-465/807 bait and with different Hinderin constructs (fig. 2). Yeast cotransformants of SMC3-465/807 and the Hinderin plasmid retrieved from the two-hybrid library (H-47/578) grew rapidly. This result was confirmed by switching the bait and prey vectors. However, the H-47/578 construct did not interact with the N- and C-terminal domains of SMC3 (SMC3-1/186 and SMC3-976/1217 respectively) or with the hinge region of SMC1 (SMC1-485/670). A Hinderin construct harboring only the protein central globular domain (H-177/360) furthermore did not interact with the SMC3 bait, thus pointing to a substantial difference in the binding properties of the hinge domain of the SMC proteins and the corresponding domain of Hinderin. On the contrary, both the N- and C-terminal domains of Hinderin (H-1/85 and H-360/578) interacted with the SMC3 hinge domain. The assay of the β-galactosidase activity expressed by the yeast as a result of the protein-protein interaction provided a quantitative measure of the rate of the process. The strongest interaction was detected between SMC3-465/807 or SMC3-465/716 and H-47/578. The Hinderin constructs H-1/85 and H-360/578 displayed a lower interaction activity suggesting that the protein N- and C-terminal domains synergically bind to SMC3. An Hinderin construct (H-64/360), consisting of the central globular domain in addition to part of the protein N-terminal domain, displayed binding activity similar to that of the N-terminal globular domain alone (H-1/85). The protein central globular domain therefore does not affect the binding rate of Hinderin to SMC3. Truncation mutants of SMC3-465/807 were generated to map the region of SMC3 responsible for the interaction with Hinderin. When tested only SMC3-465/716 gave origin to colonies growing at the same rate as observed with the SMC3 bait (fig. 2). The remaining constructs, in which the SMC3 hinge region had been partially or completely deleted, produced no colonies. The intact SMC3 hinge domain is thus required for the interaction with Hinderin. Similarly the interaction between SMC3-465/807 and the hinge region of SMC1 (SMC1-485/670) gave strong reactivity whereas truncation mutants of SMC3 lacking part of the cohesin protein central globular domain (SMC3-552/807 and SMC3-465/643) did not interact. This finding is consistent with previous reports showing that SMC3 and SMC1 dimerization occurs through interaction of the terminal regions of the hinge domains of the two proteins [7,14].

### Hinderin interacts with SMC3 in vivo and is a binding competitor for SMC1

**Figure 3:**
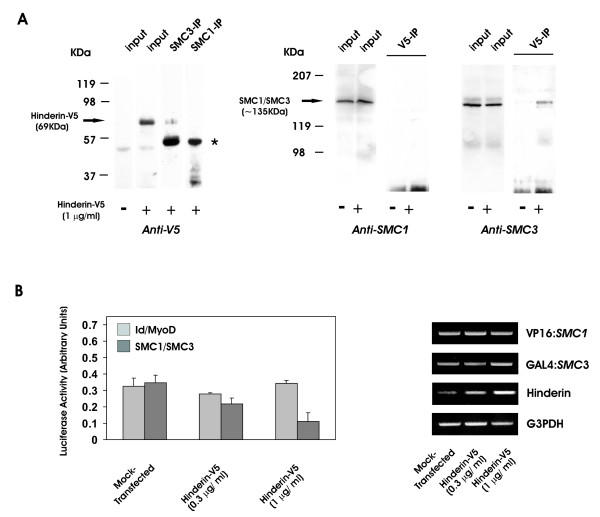
**Protein coimmunoprecipitation and competitive binding of Hinderin to SMC3. **A) To monitor the Hinderin interaction, 293 cells were transfected with 1 μg/ml of Hinderin-V5 expression vector and incubated for 48 h. Mock transfected cells served as control. For SMC1 and SMC3 coimmunoprecipitation experiments, cell lysates (500 μl) were incubated with either 25 μg of anti-SMC1 or anti-SMC3 antibody for 2 h at RT. The immunocomplexes were then captured on agarose-protein G and analyzed by electrophoresis on 12% SDS-PAGE. After transblotting to a nitrocellulose membrane, Hinderin-V5 was identified using an anti-V5 monoclonal antibody and HRP-conjugated secondary antibody. The migration position of the Hinderin-V5 fusion protein present in the input cell lysate (200 μl) and in the immunoprecipitate is indicated by an arrow. Goat immunoglobulins are identified by an asterix. The presence of SMC1 and SMC3 in the Hinderin-V5 immunoprecipitate was assessed by incubation of cell lysates (500 μl) with 10 μg murine anti-V5 antibody. The immunocomplexes absorbed on agarose-protein G were analyzed on 8% SDS-PAGE and immunoblotted with either anti-SMC1 or anti-SMC3 antibody. SMC1 and SMC3 immunoblots of the input material (200 μl) are also illustrated. B) The effect of Hinderin on the interaction between the SMC3 and SMC1 hinge domains was investigated in 293 cells expressing different level of Hinderin by using a mammalian two-hybrid assay system. SMC3-474/702 and the SMC1-474/663 hinge domains in pBIND and pACT vectors respectively, were cotransfected in 293 cells together with Hinderin-V5 expression vector (0.3 or 1 μg/ml) or alternatively 1 μg/ml of the empty pcDNA3.1 vector (mock-transfected) and the reporter pG5/luc vector using Lipofectamine. Cells transfected with pBIND-Id and pACT-MyoD fusion protein expression vectors were used as control to assess the specificity of the Hinderin effect on protein-protein interaction. Luciferase activity was assayed after 48 h. The bars represent the mean ± SD of the values (n = 3). Semiquantitative RT-PCR was employed to assess the transcript level of GAL4:SMC3 and VP16:SMC1 fusion proteins and of G3PDH in cells ectopically expressing different levels of Hinderin.

In order to investigate whether Hinderin associates with SMC3 in vivo, 293 cells were transiently transfected with 1 μg/ml of Hinderin-V5 expression vector and incubated 48 h. The transfected cells displayed three-fold elevation of the *Hinderin *transcript level but SMC1 and SMC3 expression was not affected (fig. 3A, V5-IP input lanes and fig 3B, RT-PCR results). After addition of anti-SMC3 or alternatively anti-SMC1 antibody to the cell lysate, the immunocomplexes were analyzed on SDS-PAGE followed by Western-immunoblotting using anti-V5 antibody (fig. 3A). This revealed the presence of Hinderin-V5 in the SMC3 immunoprecipitate. On the contrary, Hinderin-V5 was not detected in the SMC1 immunoprecipitate, thus corroborating the results of the yeast two-hybrid system interaction experiments. Furthermore, SMC3 but not SMC1 could be recovered in Hinderin-V5 immunocomplexes. In order to examine whether the interaction between SMC1 and SMC3 is affected by Hinderin concentration in vivo, experiments were conducted in 293 cells expressing increasing levels of Hinderin and by assessing the rate of SMC1-SMC3 interaction using a mammalian two-hybrid system (Promega Checkmate). In this assay the interaction between the GAL4:SMC3 activating domain fusion protein and the VP16:SMC1-binding domain fusion protein activates the expression of the *firefly *luciferase encoded by a reporter vector (pGL5). The rate of interaction between SMC3 and SMC1 is thus directly proportional to the level of luciferase that is expressed. We found that transfection of 293 cells with increasing amount of the Hinderin-V5 expression vector caused a dose-dependent decrease of luciferase activity. In cells overexpressing Hinderin, the interaction between the cohesin proteins was gradually reduced down to ~34% (n = 3, p < 0.05) (fig. 3B). Ectopic expression of Hinderin had no effect on the expression of the bait and prey fusion proteins as determined by RT-PCR, using primers encompassing the coding sequence of fusion proteins. The results are consistent with the notion that Hinderin competes with SMC1 for binding to SMC3, thereby negatively affecting the interaction between the two cohesin proteins.

## Discussion

**Figure 4:**
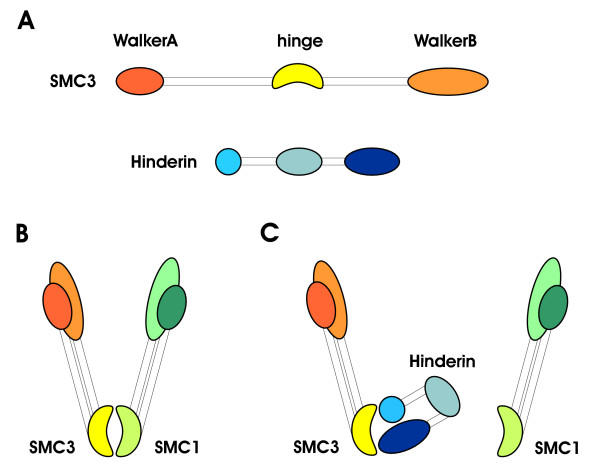
**Proposed model of interaction of Hinderin with SMC3. **A) The five-domain structure of Hinderin is compared to that of SMC3. The different structural domains are drawn in scale to allow a direct comparison of the size of the terminal globular regions, the two coiled-coil domains and the central globular domain in the two proteins. B) Mode of interaction of the SMC1-SMC3 dimer. The juxtaposition of the cohesins hinge domains interacting through sites located at the globular-coiled coil domain boundaries (ref. 6) is illustrated. C) Postulated mechanism for the competitive binding of Hinderin to the hinge domain of SMC3. The N- and C-terminal globular domains of Hinderin are shown to interact with binding sites located on the SMC3 hinge. The Hinderin central globular domain is not involved in the binding to SMC3 but may play a role by orienting the N- and C-terminal globular domains toward their targets.

In eukaryotic cells, the disjunction of the sister chromatids at the onset of anaphase is required to maintain chromosomal stability and to prevent aneuploidy [12,15]. Human somatic cells with unrestrained separase activity display retarded sister separation and increased rate of chromosomal breakage [10]. On the other hand, the overexpression of Scc1 in mouse fibroblasts inhibits the cells proliferation, implicating this protein and its complex with the other cohesin components in the control of mitotic cell cycle progression [16]. We have previously reported that overexpression of SMC3 has cell transforming potential [17] and we have determined that SMC3 level is controlled in intestinal epithelial cells through the APC/β-catenin/TCF4 transactivation pathway [18], a signaling system that is almost invariably altered in colon carcinomas. These findings support the idea that alteration of the level of the components of the cohesin complex has important consequences that may trigger a tumorigenic cascade. A key event in the formation of the cohesin multimeric complex is the dimerization of SMC1 with SMC3 which occurs through the interaction between the two proteins central globular domains. The hindrance of this process is likely to have a significant effect on the formation and the function of the cohesin complex and on the maintenance of a stable chromosome population. Given this postulate, we have screened a human cDNA library to identify proteins interacting with the hinge region of SMC3. Clones coding for Hinderin accounted for 15% of those identified in this screening. Interaction between Hinderin and SMC3 has been further confirmed by mapping the site of binding, in co-immunoprecipitation experiments using cell lysates, and in a two-hybrid mammalian system. Furthermore we have determined that SMC1 and SMC3 interaction rate is inversely related to the level of Hinderin expressed by mammalian cells. Hinderin displays the same five-domain structural organization of the SMC family [19]. However, the central globular domain of the protein does not appear to be involved in the binding with SMC3. Protein-protein interaction studies with a set of truncation constructs are rather consistent with the conclusion that SMC3 specifically interacts with the Hinderin terminal globular domains. An interaction model that is plausible with the protein-protein interaction results is illustrated in fig 4. The model predicts the occupancy of the hinge region of SMC3 by the two terminal globular domains of Hinderin that come in close proximity by virtue of the flexibility of the protein central globular domain. In SMC1 and SMC3 the coiled-coil domains are in antiparallel orientation and by interacting they allow the N- and C-globular domains to join and form a functional ATPase head that interacts with Scc1 [19]. Given the similarity with the structural organization of the SMC proteins and the interaction modality with SMC3, the Hinderin coiled coil domains might contribute to the binding of the N- and C-terminal globular domains to SMC3.

## Conclusions

In summary, we have identified a novel interacting partner of SMC3. The protein, named Hinderin, specifically interacts with the hinge domain of SMC3. The protein is ubiquitously expressed in human tissues. We speculate that when in a certain context, SMC3 association with Hinderin becomes favored compared to the association to SMC1, the availability of SMC3 to engage in the cohesin complex formation is reduced. The binding of SMC3 to proteins affecting its association to functional partners represents a new modality of regulation of SMC3 activity.

## Methods

### Screening of a human cDNA library by yeast two-hybrid system

In order to identify proteins encoded by the human fetal brain cDNA library and interacting with the hinge domain of SMC3, a bait plasmid was generated by subcloning the SMC3-465/807 polypeptide coding region into the yeast two-hybrid system bait vector pGBKT7 (Clontech) (see fig. 2 for a diagram of the constructs used and their designation). The insert was generated by PCR using *Pfu *DNA polymerase and primers terminated with restriction sites that allowed the directional cloning of the products into the accepting vector (see Additional file 1 for a listing of the primers used in this study). Mouse full-lenght SMC3 cDNA was used as template. To identify the gene encoded by the interacting plasmid, the prey insert was sequenced by priming at the Gal4AD site (5'-AATACCACTACAATGGA-3'). The sequences were BLASTed against the nr and human dEST databases. DNA restriction digestions provided information on the size of the clones retrieved.

### 5' RACE and cloning of the complete Hinderin coding sequence

The total RNA was isolated using TRI-reagent from 293, HepG2 and HeLa cells. The 5' RACE assay was performed using a RLM-RACE Ambion kit. The generated cDNA was used as template for nested PCR. The inner PCR product was cloned into pCRII-Topo vector (Invitrogen) and sequenced using a T7 primer. The sequence matched that of the DKFZp451C1618 clone (AL832625) and extended 5' to the published sequence of KIAA1328 (AB037749). Based on this information, the complete *Hinderin *coding region was generated by RT-PCR utilizing mRNA extracted from 293 cells, and the product cloned in frame with the tag sequence in pcDNA3.1/V5-His TOPO (Invitrogen). When transfected into 293 cells, the expressed product detected with an anti-V5 monoclonal antibody had size 69 KDa, as expected for a protein encoded by the Hinderin-V5 fusion gene (fig. 3A).

### Mapping of the protein interacting sites

In order to map the SMC3 interacting site(s) a series of truncated constructs were produced in pGBKT7 vector. The inserts required for the SMC3-1/186, SMC3-976/1217, SMC3-552/807 and SMC3-711/807 constructs were produced by PCR. SMC3-465/550 and SMC3-465/716 were generated by introducing stop codons in the sequence of SMC3-465/807 using a QuikChange XL kit (Stratagene). The mutated duplex oligonucleotides used had the forward sequence: 5'-CTTTCTATACTTGTGT AAGTCACTGCTGGTAAC-3' and 5'-GACCAGTTGATGAACTAAATGCAGATAGAG-3', respectively. SMC3-465/643 and SMC3-643/807 were obtained by digesting SMC3-465/807 at the *Pst*I or alternatively the *Nde*I restriction sites present in the vector multiple cloning site and at the *Sma*I site of the insert. The resulting linear constructs were blunt-ended and religated. pGADT7-SMC3-465/807 was generated by retriving the insert from the bait vector. SMC1-485/670 encoding the entire SMC1 hinge region, was generated by RT-PCR from 293 cells mRNA and cloned in pGADT7. Hinderin deletion constructs H-64/360 and H-360/578 were generated by restriction digestion of the pACT2-Hinderin-47/578 clone identified in the yeast two-hybrid system screening with *Nco*I/*Bgl*II and *Bgl*II/*Xho*I respectively, followed by religation of the blunt-ended vector. Hinderin in bait pGBKT7 vector was generated by retrieving the H-47/578 insert from the prey clone. H-177/360 and H-1/85 inserts were obtained respectively by PCR and by digestion of the Hinderin pcDNA3.1 expression vector with *BamH*I/*EcoR*I. Both were subsequently subcloned in pGADT7. To assess the strenght of interaction between different SMC3 and Hinderin domains, three colonies were randomly selected and grown overnight in 5 ml of selection media. After cell lysis by freeze thawing in 300 ml of 100 mM Na_2_HPO_4 _pH 7.0, 10 mM KCl, 1 mM MgSO4, buffer, β-galactosidase activity was assessed using ortho-nitrophenyl-β-D-galactopyranoside as substrate (1 mM) in the presence of 50 mM β-mercaptoethanol. After 2 h incubation at 37°C the color intensity was read at 420 nm.

### Northern blot hybridization and semiquantitative PCR

Total mRNA was extracted with TRI-reagent from subconfluent HeLa and HCT116 cells and separated on 1% agarose. After transfection to a nitrocellulose filter, the *Hinderin *transcript was identified by hybridization to a 335 bp ^32^P-labeled cDNA probe annealing to the 5'-end region of the gene. In order to examine the expression of *Hinderin *in different human tissues, semiquantitative RT-PCR was performed by using 0.5 μg of Marathon-ready first strand cDNA (Clontech) from 16 tissues. The PCR reaction was monitored at 20 and 30 cycles and the product analyzed on 2% agarose. In order to normalize for possible differences in mRNA content, the expression of the housekeeping gene G3PDH was analyzed in each sample.

### Protein secondary structure prediction and identification of orthologue forms of Hinderin in other species

The human polypeptide sequence was analyzed with the COILS program to predict globular and coiled-coil domains. The scanning window was set at 21. The homology with other known protein family was examined by querying the NCBI Conserved Domain protein database. We used PSORT to scan for nuclear and other localization signal consensus sequences. To identify orthologue forms of *Hinderin *in other species, the human protein sequence was BLASTed against the translated EST database of m. fascicularis, mouse, rat, cow, sheep, dog, zebrafish, c. elegans, drosophila, and s. cereviasie. When a homologous sequence had been identified in lower organisms, we ran the COILS program to assess whether it displayed the same secondary structure as that of the matching human sequence.

### Protein complex immunoprecipitation

293 cells were grown at ~80% confluence in 35 mm cm plates and transfected with 1 μg of Hinderin-V5 expression vector. After 48 h of incubation, the cells were washed in ice-cold phosphate-buffered saline and lysed in 1.2 ml of 50 mM Tris-HCl, pH 7.5, 150 mM NaCl, 1% Nonidet P40, 0.5% Na-deoxycholate buffer containing 100 mM NaF, 2 mM Na_3_VO_4_, and a cocktail of protease inhibitors. The cell lysate was centrifuged at 12,000 g and the recovered supernatant preabsorbed on protein G-agarose. Aliquots (500 μl) of the cell lysate were then incubated overnight at 4°C with either 25 μg of goat anti-human SMC3 antibody or goat anti-human SMC1 antibody (Santa Cruz Biotech) or alternatively with 10 μg mouse anti-V5 antibody (Invitrogen). The immunocomplexes were captured on Protein G-agarose by incubating 1 h at 4°C. After washing in immunoprecipitation buffer containing 300 mM NaCl, the protein immunocomplex was analyzed by SDS-PAGE and the proteins transferred to nitrocellulose membranes by electroblotting. After saturation in 4% dry milk/0.1% Tween 20 in PBS, the filter was incubated 1 h at RT with primary antibody. The anti-SMC1 and anti-SMC3 immunoprecipitate filter blots were incubated with anti-V5 monoclonal antibody (200 ng/ml) whereas the V5 immunoblots were incubated with either anti SMC1 (100 ng/ml) or anti-SMC3 (100 ng/ml) antibodies. After incubation with species-specific anti IgG HRP-conjugated secondary antibody (1:10,000), the immunocomplexes were visualized by enhanced chemiluminescence reaction (ECL).

### Mammalian two-hybrid interaction assay

Inserts corresponding to the SMC3-474/702 and SMC1-474/663 hinge domains were generated by PCR and ligated in the pBIND or the pACT vectors (Promega) respectively through ligation at the *BamH*I and *Sal*I sites. The resulting bait and prey DNA constructs (0.25 μg/ml each) together with the Hinderin-V5 expression vector (either 0.3 or 1 μg/ml or alternatively 1 μg/ml of empty pcDNA3.1 vector) and the reporter plasmid pG5/luc encoding the *firefly *luciferase (0.1 μg/ml), were co-transfected into 293 cells using Lipofectamine. Control experiments were conducted using pACT-Id and pBIND-MyoD vectors (Promega) encoding respectively GAL4:Id and VP16:MyoD fusion proteins known to interact in vivo [20]. After 48 h incubation, cells were lysed and the expressed luciferase activity quantitated using a dual luciferase reporter assay kit (Promega) and a Lumat LB 9501 luminometer. *Firefly *luciferase values were corrected for the transfection efficiency using the values of the *Renilla *luciferase activity encoded by the pBIND vector under the control of a strong constitutive promoter. In order to assess the effect of Hinderin on the bait and prey expression, total RNA was extracted from a group of transfected cells and the specifc transcript levels quantitated by RT-PCR using primers annealing to the ends of the fusion protein coding sequence.

## Authors' contributions

CAP carried out the two-hybrid system experiments including the immunoprecipitation studies, generated the necessary DNA constructs, and performed the gene expression analysis. GG conceived and coordinated the studies and drafted the manuscript.

## Supplementary Material

Additional File 1 **Primer and adapter sequences **oligonucleotide sequence Click here for file
