# Supplementary material for: Hinderin, a five-domains protein including coiled-coil motifs that binds to SMC3
Source: BMC Cell Biol. 2005 Jan 18;6:3. doi: 10.1186/1471-2121-6-3 (PMC547899; doi:10.1186/1471-2121-6-3)
Supplement: Additional File 1 — Primer and adapter sequences oligonucleotide sequence [file 1471-2121-6-3-S1.doc]

# Yeast two-hybrid system bait and prey primers

**SMC3/1-186**: 5’-­CATATGTACATCAAGCAGGTGATCATC-3’

5’-GGATCCCGTTTGCCCTCTGTTTCTTT-3’

**SMC3-465/807:** 5’-GATGAATTCCAAAGTGAAAGAAATTACTTGTGG-3’

5’-CTGCTGCAGCTGACGGATTTCATC-3’;

**SMC3-976/1217:** 5’-­ACAGAATTCAAGAAGTACAGCCACGTGAACAAA-3’

5’-TCTCTGCAGTGATCACATCAA TGTGACTAACCT-3’

**SMC3-552/807:** 5’-GTGGAATTCACTGCTGGTAACAGGTTATTTTAT-3’

5’-CTGCTGCAGCTGACGGATTTCATC-3’

**SMC3-711/807:** 5’-AATGAATTCGACCAGTTGATGAACCAAATGCAG-3’

5’- CTGCTGCAGCTGACGGATTTCATC-3’

**HIND-1/578:** 5’-TTCAAGATGGCGGACGTGGCG-3’

5’-AATGAAAAATATATCTTCCAGAATCTGATTCTC-3’

**HIND-177/360:** 5’-TCACCATGGCTCTCTCAGAACTT-­3’

5’-CTATCTTCTGAGATCTGCTTTTTCAAA-3’

**SMC1-485/670:** 5’-AGCATATGCCCGCATCGACCGCCAGG-3’

5’-­TCGGATCCCAGCGCCGTGCCTTGGC-3’

# Mammalian two-hybrid system bait and prey primers

**SMC3-474/702:** 5’-GTGGATCCAGGAGAATGCAGAACAGCAAGCA-3’

5’-GGTGTCGACCTGCTGCATTTGGTTCATCAACTG-3’

**SMC1-474/663:** 5’-ATGGGATCCATAAGGAGCTGAACCAGGTGATGGAG-3’

5’-TCCGTCGACTGCCACTGTCTTGTGGCGCTG-3’

**5’-RACE adapter and primers**

**Adapter:** 5’-GCTGATGGCGATGAATGAACACTGCGTTTGCTGGTT

TGATGAAA-3’

**Outer primers:** 5’-GCTGATGGCGATGAATGAACACTG-3’

5’-GCTTAGAAGTTCTTGGCATTCTCTATACT-3’

**Inner primers:** 5’-CGCGGATCCGAACACTGCGTTTGCTGGCTTTGATG-3’

5’-CTTCTTCTCAAATGACTCCTGCTCA-3’

**Semiquantitative RT-PCR primers**

**Hinderin:** 5’-GTTAAACTTAAGACTTCCAGGGTGACT-3’

5’-GCTTAGAAGTTCTTGGCATTCTCTATACT-3’

**VP16:smc1** 5’-GCTACTGTCTTCTATCGAACAAGC-3’

5’-TTCAGGTCACTGGCCCCAC-3’

**GAL4:SMC3** 5’-GCTACTGTCTTCTATCGAACAAGC-3’

5’-CTTCGTGCTGACTTCATCTGAATC-3’

**g3pdh:** 5’-TGAAGGTCGGAGTCAACGGATTTGGT-3’

5’-CATGTGGGCCATGAGGTCCACCAC-3’
